# Alterations of the Gut Microbiota in Patients With Severe Chronic Heart Failure

**DOI:** 10.3389/fmicb.2021.813289

**Published:** 2022-01-31

**Authors:** Weiju Sun, Debing Du, Tongze Fu, Ying Han, Peng Li, Hong Ju

**Affiliations:** ^1^Department of Cardiology, The First Affiliated Hospital of Harbin Medical University, Harbin, China; ^2^Beidahuang Industry Group General Hospital, Harbin, China; ^3^Harbin Medical University, Harbin, China; ^4^Department of Cardiology, The Fourth Affiliated Hospital of Harbin Medical University, Harbin, China; ^5^National Center for Biomedical Analysis, Beijing, China; ^6^Heilongjiang Vocational College of Biology Science and Technology, Harbin, China

**Keywords:** severe chronic heart failure, gut microbiota, 16S rRNA gene, SCFA, patients

## Abstract

Chronic heart failure (CHF) is the final outcome of almost all forms of cardiovascular diseases, remaining the main cause of mortality worldwide. Accumulating evidence is focused on the roles of gut microbial community in cardiovascular disease, but few studies have unveiled the alterations and further directions of gut microbiota in severe CHF patients. Aimed to investigate this deficiency, fecal samples from 29 CHF patients diagnosed with NYHA Class III-IV and 30 healthy controls were collected and then analyzed using bacterial 16S rRNA gene sequencing. As a result, there were many significant differences between the two groups. Firstly, the phylum *Firmicutes* was found to be remarkably decreased in severe CHF patients, and the phylum *Proteobacteria* was the second most abundant phyla in severe CHF patients instead of phylum *Bacteroides* strangely. Secondly, the α diversity indices such as chao1, PD-whole-tree and Shannon indices were significantly decreased in the severe CHF versus the control group, as well as the notable difference in β-diversity between the two groups. Thirdly, our result revealed a remarkable decrease in the abundance of the short-chain fatty acids (SCFA)-producing bacteria including genera *Ruminococcaceae UCG-004*, *Ruminococcaceae UCG-002*, *Lachnospiraceae FCS020 group*, *Dialister* and the increased abundance of the genera in *Enterococcus* and *Enterococcaceae* with an increased production of lactic acid. Finally, the alternation of the gut microbiota was presumably associated with the function including Cell cycle control, cell division, chromosome partitioning, Amino acid transport and metabolism and Carbohydrate transport and metabolism through SCFA pathway. Our findings provide the direction and theoretical knowledge for the regulation of gut flora in the treatment of severe CHF.

## Introduction

Chronic heart failure (CHF) is a major health problem worldwide. It is the final outcome of almost all forms of cardiovascular diseases. CHF is recognized not only as a deregulation of hemodynamic disorder and neurocrine activation, but also an uncontrolled elevation of inflammatory responses and oxidative stress (Anker and Von Haehling, 2004; Gajarsa and Kloner, 2011; Hage et al., 2017; Huang et al., 2020). CHF is still associated with a high rate of hospitalization and a devastating prognosis, despite the recent development of modern combinational therapeutic strategies. Therefore, it is possible that important pathogenic mechanisms have not been targeted by current treatments, such as gut microbiota dysbiosis which have also been implicated to play a role in the development of cardiovascular diseases, including CHF (Brial et al., 2018; Jia et al., 2019; Cheng et al., 2020; Huang et al., 2020; Sanchez-Rodriguez et al., 2020).

The gut microbiota, comprising the trillions of bacteria in the gastrointestinal tract, is essential for maintaining human health in many aspects, digesting the indigestible nutrients of the host, producing vitamins and hormones, shaping the development of the mucosal immune system, and preventing the colonization of pathogenic bacteria (Amoroso et al., 2020; Sanchez-Rodriguez et al., 2020; Deledda et al., 2021). Host-microbiota interactions involving inflammatory and metabolic pathways have been proposed to contribute to the pathogenesis of CHF (Dantzer et al., 2018; Moshkelgosha et al., 2018; Cheng et al., 2019, 2021; Zhang et al., 2019; Kwon et al., 2020). In recent years, several sequencing-based studies have reported that the composition and function of intestinal flora between HF patients and healthy subjects are different. There are some common findings, but also considerable differences between studies (Kamo et al., 2017; Luedde et al., 2017; Cui et al., 2018; Kummen et al., 2018; Mayerhofer et al., 2018, 2020; Iqubal et al., 2020; Khan et al., 2020; Qi et al., 2021). Some computational methods have been applied in the field and other biological data (Long et al., 2021; Lv et al., 2021; Yang et al., 2021). Thus, more studies are still needed to provide detailed information on variations of gut microbial composition and its impacts on CHF, especially the severe CHF.

In order to define a more robust HF-related gut microbiota signature, we conducted this cross-sectional cohorts investigation. In this study, we collected stool samples from severe CHF patients and healthy controls, amplified the variable region of intestinal bacteria 16S rRNA, constructed a DNA library, and then assessed the taxonomic composition of the gut microbiota in patients with severe CHF.

## Materials and Methods

### Study Population and Sample Collection

Chronic heart failure patients (*n* = 29) were recruited from the First Affiliated Hospital of Harbin Medical University and the Fourth Affiliated Hospital of Harbin Medical University between April 2020 and August 2020, as well as 30 asymptomatic persons undergoing physical examinations as healthy controls. Patients who were recruited represent multiple stages of HF progression, as defined by NYHA Class III-IV. The inclusion criteria were as follows: (1) the subjects had not received antacids, probiotics, antibiotics, or antimicrobial agents within 30 days before sample collection; (2) there was no organic disease of the digestive system; and (3) they had no gastrointestinal surgery. NYHA classification was performed by patients’ treating cardiologist and adjudicated by 2 HF specialists who were blinded to the results. All patients with HF were treated according to current HF management guidelines. Associated clinical information was collected from electronic medical records. All participants (or their direct relatives) gave written informed consent, and the First Affiliated Hospital of Harbin Medical University and the Fourth Affiliated Hospital of Harbin Medical University approved all study protocols.

We collected fresh fecal samples (each 2–5 g) from all the participants 1–2 days after admission, then transferred into sterile collecting pipes and frozen at −80°C immediately.

### DNA Extraction and 16S rRNA Gene V3-V4 Region Sequencing

The bacterial DNA was extracted from the fecal samples using the Tiangen stool mini kit (Tiangen, Beijing, China) according to the manufacturer’s instructions. The extracted DNA from each sample was used as the template to amplify the V3–V4 region of 16S rRNA genes using PCR. PCR amplification, sequencing of the PCR amplicons and quality control of raw data were performed. A sequencing library of the V3–V4 regions of the 16S rRNA gene was prepared as described previously (Han et al., 2021). The purified products were mixed at an equal ratio for sequencing using an Illumina MiSeq system (Illumina Inc., United States).

### Statistical Analysis

We evaluated the quality of sequencing data using the Fast-QC software^1^ firstly. Next we obtained the clean data for subsequent analysis after removing the Chimera Sequence using QIIME2.^2^ Third, operational taxonomic units (OTUs) were delineated at the cutoff of 97% also using QIIME2, and the sequencing results were compared and analyzed to obtain the family and genus annotations of OTUs based on the Silva database.^3^ Then α- and β-diversity analyses were performed using QIIME2 fourthly. Shannon-wiener diversity index, Simpson diversity index, the observed OTUs, PD (phylogenetic diversity)-whole-tree and Chao1 index were evaluated. A normalized OTU abundance table was used for the β-diversity analysis, including principal coordinate analysis (PCoA) based on weighted UniFrac, and unweighted UniFrac distances. Next, we performed Lefse analysis to clarify the dominant bacteria. LEfSe is a software for discovering high-dimensional biomarkers and revealing genome characteristics. LEfSe uses linear discriminant analysis (LDA) to estimate the impact of the abundance of each component (species) on the difference effect. At last, the gene function of the sample was inferred based on the species composition obtained by sequencing, and the functional difference between different groups was analyzed using PICRUSt.^4^ Subsequently, the Welch’s *t*-test method of two groups was performed using the STAMP software to filter the parts with *P*-value > 0.05, and Heatmap Plot, PCA plot, and Extented error bar graphs were drawn to reveal significant differences in species abundance between different samples.

## Results

### Baseline Characteristics

The baseline characteristics of all the participants are shown in Table 1. Patients with CHF were characterized by a greater number of males, increased prevalence of comorbidity with Atrial fibrillation, worsened cardiac functions including larger left ventricular end diastolic diameter (LVEDD), decreased left ventricular ejection fraction (LVEF) and stroke volume (SV), increased E/e’, coupled with increased serum Troponin I (TnI) and NT-pro B-type natriuretic peptide (NT-proBNP) levels. Patients with CHF also have worse renal function. Most (21) of the patients were classified to be heart failure patients with reduced ejection fraction, only 1 were classified to be heart failure patients with preserved ejection fraction, and the rest 8 of the patients were classified to be heart failure patients with midrange ejection fraction. All patients with HF were treated according to current HF management guidelines, including diuretics, βblocker, Aldosterone receptor antagonist, Angiotensin converting enzyme inhibitor/Angiotensin receptor antagonist/Angiotensin receptor neprilysin inhibitor, and Sodium-glucose cotransporter 2 inhibitor.

**TABLE 1:** Baseline characteristics of the study participants.

Variables	HF patients (*n* = 29)	Healthy controls (*n* = 30)	*P* value
Age, years	60.69 (11.67)	60.0 (9.64)	0.8062
Sex, male	24 (83%)	10 (33%)	<0.0001
BMI (kg/m^2^)	24.0 (3.47)	24.9 (3.08)	0.2849
NYHA class (III/IV)	10/19	—	—
HFrEF/HFpEF/HFmrEF	21/1/8	—	—
Hypertension	14 (48%)	11 (37%)	0.3757
Diabetes	10 (34%)	5 (16.7%)	0.1202
Atrial fibrillation	10 (6.7%)	0 (0)	0.0003
Smoking	12 (41.4%)	6 (20%)	0.0769
**Echocardiographic parameters**			
LVEDD, mm	62.0 (8.67)	43.7 (3.99)	<0.0001
LVEF, %	33.8 (9.1)	63.2 (4.65)	<0.0001
SV, ml	46.7 (11.1)	68.9 (11.94)	<0.0001
E/e’	19.3 (6.5)	13.4 (3.1)	<0.0001
**Laboratory parameters**			
TnI, ng/dL	55.87 (0.10–88.7)	0.012 (0–0.048)	0.0007
NT-proBNP, pg/mL	4745.7 (1130–16755)	124.0 (25–258)	<0.0001
Leukocyte,10^9^/L	7.2 (3.00)	6.7 (1.74)	0.4366
Neutrophils,10^9^/L	4.9 (2.51)	4.0 (1.30)	0.0878
Lymphocytes, 10^9^/L	1.63 (0.61)	2.1 (0.65)	0.0110
Monocyte, 10^9^/L	0.5 (0.18)	0.4 (0.14)	0.0632
Hemoglobin, g/L	140.7 (26.53)	140.8 (17.04)	0.9834
BUN, mg/dl	8.0 (3.04)	5.5 (1.81)	0.0003
Serum creatinine, mg/dl	87.4 (35.16)	67.4 (18.35)	0.0078
Fast glucose	6.5 (3.67)	5.2 (1.30)	0.0698
Cholesterol	4.2 (0.96)	5.0 (0.90)	0.0013
Triglycerides	1.5 (0.92)	1.9 (1.66)	0.2305
HDL-C	0.9 (0.22)	0.91 (0.22)	0.8917
LDL-C	2.5 (0.87)	2.9 (0.80)	0.0592
Treatment diuretics	29 (100%)	—	—
β blocker	27 (93%)	—	—
MRA	29 (100%)	—	—
ACEI/ARB/ARNI	26 (90%)	—	—
SGLT2i	21 (72%)	—	—

*Results are presented as median (with standard error or upper and lower quartiles) or% where appropriate. BMI, body mass index; NYHA, New York Heart Association; HFrEF, heart failure with reduced EF; HFpEF, heart failure with preserved EF; HfmrEF, heart failure with midrange EF; LVEDD: left ventricular end diastolic diameter; LVEF: Left ventricular ejection fraction; TnI, Troponin I; NT-proBNP: NT-pro B-type natriuretic peptide; HDL-C, high density lipoprotein-cholesterol; LDL-C, low density lipoprotein-cholesterol; MRA, Aldosterone receptor antagonist; ACEI, Angiotensin converting enzyme inhibitor; ARB, Angiotensin receptor antagonist; ARNI, Angiotensin receptor neprilysin inhibitor; SGLT2i, Sodium-glucose cotransporter 2 inhibitor.*

### Species Classification

**FIGURE 1:**
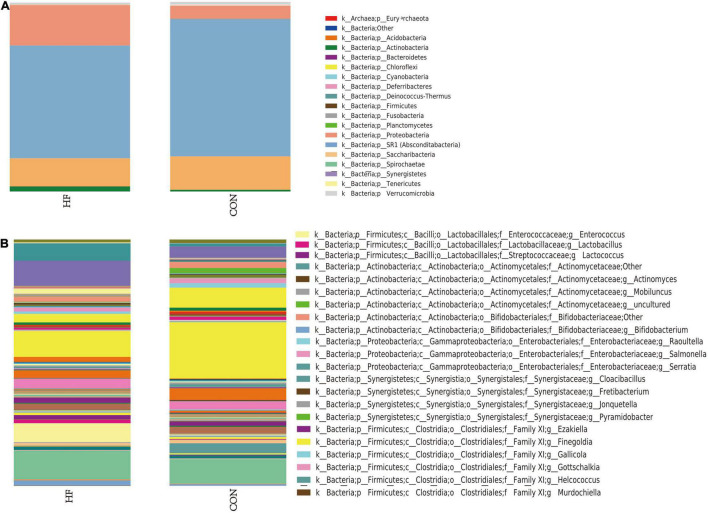
The distribution of relative abundance in the CHF and the healthy control group. Panel **(A)** shows the distribution of relative abundance of top 19 at phylum level in the two groups. Panel **(B)** shows the distribution of relative abundance at the genus level in the two groups. HF, CHF group; CON, the healthy control group.

The different distribution of relative abundance of top 19 at the phylum level in the two groups is presented in Figure 1A. Sequencing analysis showed that gut microbiota of the two groups were mainly classified into four phyla, including the phyla *Firmicutes*, *Proteobacteria*, *Bacteroidetes*, and *Actinobacteria*. The phylum *Firmicutes* was found with the highest abundance of reads in CHF patients, accounting for 59.5% in total, which was significantly decreased versus that of an abundance of 72.4% in the controls. The second was the phylum *Proteobacteria*, accounting for 21.3% in total, which was much more abundant versus that of an abundance of 6.9% in the controls. Likewise, bacteria belonging to the phyla *Actinobacteria* were more abundant in CHF patients than that in the healthy controls (2.7 vs. 0.9%), While bacteria belonging to the *Bacteroidetes* phyla were slightly less abundant in HF patients than that in the healthy controls (14.9 vs. 17.7%).

At the genus level, the microflora of CHF patients was characterized by less abundant of *Faecalibacterium* (10.5 vs. 22.8%), as well as more abundant of *Escherichia-Shigella* (10.3 vs. 4.6%), *Enterococcus* (7.7 vs. 0.0%), and *Klebsiella* (6.9 vs. 1.1%) than that in the healthy controls (Figure 1B).

### Analysis of α and β Diversity Index

**FIGURE 2:**
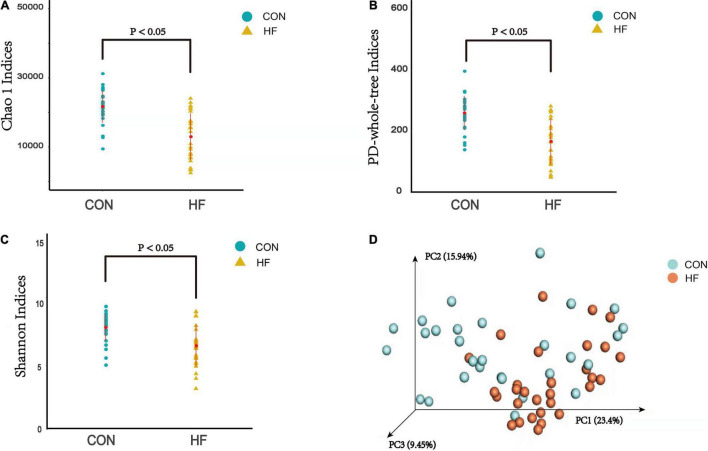
The α-diversity indices and the β-diversity of the gut microflora between the CHF group and the healthy control group. **(A)** PD-whole tree indice; **(B)** Chao1 indices; **(C)** Shannon indices; **(D)** β-diversity based on the weighted UniFrac. HF, CHF group; CON, the healthy control group.

The α diversity analysis was performed and then chao1 curve, observed-otus curve, PD-whole-tree curve, Shannon-Wiener curve and Simpson curve based on the species annotation information were subsequently obtained by sequencing analysis. As a result, the chao1 and PD-whole-tree indices were significantly decreased in the CHF versus control group, as well as the Shannon indices (Figures 2A–C). The taxonomic composition of the metagenomic populations of the gut microflora samples from patients with CHF compared to those from the healthy control group were also analyzed using Principal Coordinate Analysis (PCoA). The differences in β-diversity based on the weighted UniFrac between the HF and healthy control groups were also shown in Figure 2D, which indicates that the fecal microbial structure in the CHF group was obviously different than that of the healthy control group in condition of the presence of OTU.

### Analysis of Differential Taxonomy Expression

**FIGURE 3:**
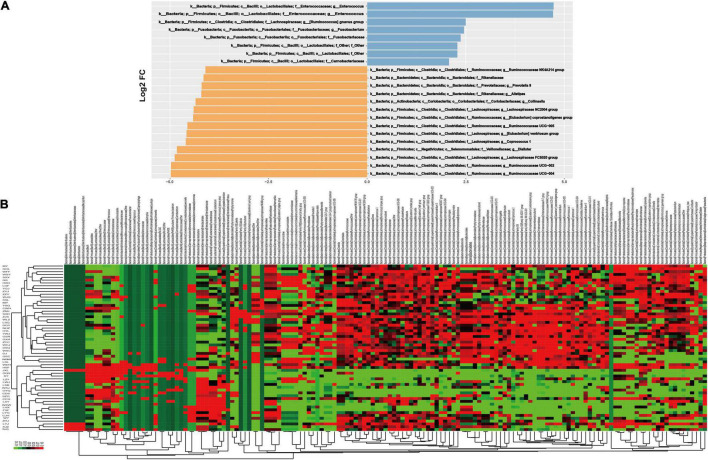
The results of differential taxonomy expression analysis using limma algorithm. Panel **(A)** shows the differential taxonomy expression in the CHF patients vs. healthy control group; **(B)** shows the differential taxonomy expression in every samples.

A differential taxonomy expression analysis was performed using limma algorithms, focusing on differences at the genus level (Figure 3). There was a remarkable difference with 152 generus in fecal microflora between the CHF and healthy control group in our result. Among these changes, the decreased abundance of the genera *Ruminococcaceae UCG-004*, *Ruminococcaceae UCG-002*, *Lachnospiraceae FCS020 group*, *Dialister* and the increased abundance of the genera in *Enterococcus* and *Enterococcaceae* were the most notable features (Figure 3B).

### Analysis of Predictive Function

**FIGURE 4:**
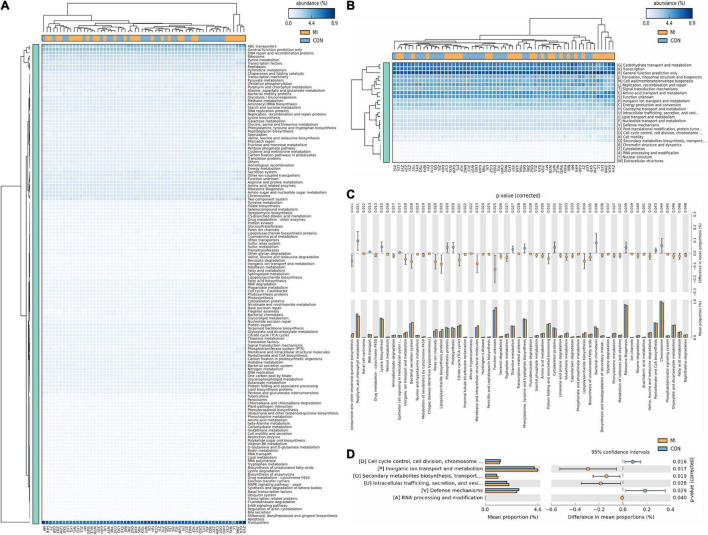
The results of PICRUst based on closed-reference OTU to predict the abundances of functional categories COG orthologs (COs) and KEGG orthologs (KOs). Panels **(A,C)** shows the KOs with significantly different abundances in the fecal microbiome between the CHF group and healthy control group; **(B,D)** shows the COs with significantly different abundances in the fecal microbiome between the CHF group and healthy control group. HF, CHF group; CON, the healthy control group.

Based on the closed-reference OUTs, PICRUSt was utilized to predict abundances of the functional category COG orthologs (COs) and KEGG orthologs (KOs). Some of these COs and KOs were indicated to be significantly different in fecal microbiomes between the CHF and healthy control group (*P* < 0.05; Figure 4). Furthermore, there were also some meaningful results related with the function including cell cycle control, cell division, chromosome partitioning, Inorganic ion transport and metabolism, translation, ribosomal structure and biogenesis, amino acid transport and metabolism and carbohydrate transport and metabolism.

## Discussion

In the current study, bacterial 16S rRNA gene sequencing was applied to confirm the composition and differential expression in gut microbiota between 29 severe CHF patients and 30 healthy controls, resulting in a number of notable differences between these two groups. Firstly, the phylum *Proteobacteria* was significantly more abundant in CHF patients than controls, whereas the phylum *Firmicutes* was found remarkably decreased in CHF patients. Secondly, the α diversity indices significantly decreased in the CHF versus control group, as well as the notable differences in β-diversity between the CHF and healthy control groups, which indicates that the fecal microbial structure in the CHF group was obviously different than that of the healthy control group in condition of the presence of OTU. Thirdly, the microflora of CHF patients was characterized by the decreased abundance of the genera *Ruminococcaceae UCG-004*, *Ruminococcaceae UCG-002*, *Lachnospiraceae FCS020 group*, *Dialister* and the increased abundance of the genera in *Enterococcus* and *Enterococcaceae*. Finally, the alternation of the gut microbiota was presumably associated with the function including cell cycle control, cell division, chromosome partitioning, inorganic ion transport and metabolism, translation, ribosomal structure and biogenesis, amino acid transport and metabolism and carbohydrate transport and metabolism. To our knowledge, this is the first study to explore the changes in the gut flora of patients with severe CHF.

Tang et al. (2017) put forward the “gut hypothesis of heart failure” for the first time. The hypothesis implies that reduced cardiac output caused by heart failure can lead to decreased intestinal perfusion, mucosal ischemia, and then intestinal mucosal destruction. These changes in the intestinal barrier function, in turn, can lead to increased intestinal permeability, intestinal malnutrition, bacterial translocation and increased circulating endotoxins, resulting in the potential inflammation associated with HF (Nagatomo and Tang, 2015; Tang et al., 2017, 2019; Harikrishnan, 2019). There are also several studies that have reported the composition and function of intestinal flora between HF patients and healthy subjects are different (Kamo et al., 2017; Luedde et al., 2017; Cui et al., 2018; Mayerhofer et al., 2018, 2020). Likewise, we found significant differences in the composition of fecal microbes between CHF patients and healthy controls, suggesting that there is a link between intestinal microflora disorders and CHF. At the phylum level, *Firmicutes* and *Bacteroides* are the two most abundant phyla in the healthy intestine. They are closely related to environmental conditions and may be beneficial or problematic for human and animal health. However, the phylum *Firmicutes* was remarkably decreased in severe CHF patients simultaneously in our study. What is more, the phylum *Proteobacteria* was the second most abundant phyla in severe CHF patients instead of phylum *Bacteroides*. All the members of the phylum *Proteobacteria* are Gram-negative bacteria, with the outer membrane mainly composed by lipopolysaccharide (LPS). Most of the phylum *Proteobacteria* are pathogenic bacteria, as a result, it is considered to be a microbial signature of dysbiosis in gut microbiota (Shin et al., 2015). Furthermore, LPS leaking into the blood through the intestinal wall can stimulate the production of a variety of pro-inflammatory cytokines, which are involved in the apoptosis, hypertrophy and fibrosis of heart cells, playing an important role in the occurrence and development of heart failure (Sandek et al., 2012).

Microbiota diversity has been considered to be a new health biomarker (Shanahan, 2010; Tang et al., 2018; Aponte et al., 2020; Nadia and Ramana, 2020; Mulpuru et al., 2021). The α diversity index mainly focuses on the number of species in a local uniform habitat, reflecting the abundance and diversity of the microbial community. As the indicators of the community richness, the chao1 and PD-whole-tree indices were significantly decreased in the CHF patients versus control group, as well as the Shannon indices, which is an important index used to estimate the microbial diversity. β-diversity is a comparative analysis of the microbial community composition of different groups of samples, constituting the overall diversity together with α diversity. These significantly different indexes in the current study revealed that loss of gut flora biodiversity is associated with CHF.

In the further differential classification expression analysis using the limma algorithm, we are more focused on the differences in the genus level. Consistent with other experimental results (Kummen et al., 2018; Mayerhofer et al., 2020), the decreased abundance of the genera *Ruminococcaceae* and *Lachnospiraceae* were also discovered in our study, which are known for their ability to synthesize short-chain fatty acids (SCFA) through the fermentation of dietary polysaccharides. In addition, we firstly reported that the decrease of genra *Dialister* and the increase of the genera in *Enterococcus* and *Enterococcaceae* were also the most notable features in CHF patients. *Dialister* is one of the most representative types of intestinal flora associated with irritable bowel syndrome (Lopetuso et al., 2018). The main products are lactic acid, acetic acid and formic acid, and those are also SCFAs. *Enterococcus* and *Enterococcaceae* (Byappanahalli et al., 2012; Gouba et al., 2020; Xie et al., 2021) are conditional pathogens, causing infections such as urinary tract infections, purulent abdominal infections, sepsis, endocarditis and diarrhea. On the other hand, their microbial preparations can enhance the activity of macrophage cells and promote the immune response. At the same time, due to their metabolism to produce lactic acid, they can form a lactic acid barrier to resist foreign pathogenic microorganisms. The above results suggested that the intestinal flora may participate in the occurrence and development of heart failure through the action of SCFAs. As we all know, SCFAs play an important role in the regulation of inflammation, which is definitely involved in the pathophysiological process of CHF. SCFAs can reduce the production of inflammatory factors by activating GPR41/43 (Li et al., 2018; Weber et al., 2018; Onyszkiewicz et al., 2019). Acetic acid may reduce the production of interleukin-6 and interleukin-8 (Sah et al., 2013; Yu et al., 2015), and butyric acid and propionic acid can reduce the production of interleukin-6 (Esquivel-Rendon et al., 2019; Yue et al., 2020). Butyrate plays an anti-inflammatory role through inducing Foxp3 + Treg cell proliferation and suppressing the generation of Th17 cells by activating G protein-coupled receptor 43 (Sivaprakasam et al., 2016; Bhaskaran et al., 2018). Other studies have also shown that SCFAs can also improve insulin sensitivity, regulate fat and muscle energy metabolism, and play an important role in the development of diabetes and obesity (Canfora et al., 2015; Muller et al., 2019).

The consequent results from analysis of predictive function using PICRUSt revealed several functional pathways involved in the relationship between gut microbiomes and CHF, including cell cycle control, cell division, chromosome partitioning, inorganic ion transport and metabolism, ribosomal structure and biogenesis, amino acid transport and metabolism and carbohydrate transport and metabolism. The occurrence and development of heart failure are inseparable from the disorders of carbohydrate metabolism, amino acids metabolism and lipid metabolism. The aforementioned differential classification expression analysis has indicated a notable reduction in SCFA-producing bacteria in patients with severe CHF. As a matter of fact, SCFAs can not only modulate the carbohydrate metabolism through activating G protein-coupled receptor and AMP-activated protein kinase, improving insulin sensitivity (Gao et al., 2009; den Besten et al., 2013), but also increasing the production of ketogenic amino acids and ketone bodies (Thevenet et al., 2016; Pujol et al., 2018), which is considered to be one of the energy sources of failing myocardium and closely related to the process of heart failure. Furthermore, as a histone deacetylase inhibitor, SCFAs can partly regulate cell proliferation, apoptosis and differentiation by inhibiting histone deacetylase, as well as exert anti-inflammatory effects (Koh et al., 2016; Makki et al., 2018; Alrafas et al., 2020).

Some limitations should be acknowledged. First, although all participants were from the same region, experienced a normal/routine lifestyle and had similar nutritional patterns, including typical Chinese diets based on carbohydrates versus high-fat diets, and participated in routine levels of general physical activity (e.g., housework and walking), we were still unable to completely account for the influence of diet on gut microbiota. Second, the enrolled cohort was a small sample size and predominantly male in patients with severe CHF. Further studies informing the generalizability of gut microbiota in patients with CHF are warranted. Third, it is also necessary to address temporality of associations between gut dysbiosis and CHF. Finally, multiple omics data, such as metabolomics and proteomics, will be warranted to confirm the suppose that SCFAs produced by the gut microbiota participating the pathophysiological processes of CHF and explore the exact mechanisms.

In conclusion, the current results firstly exhibited remarkable differencesion the composition and diversity of the gut flora of severe CHF patients and healthy controls using bacterial 16S rRNA gene sequencing. The microflora of severe CHF patients was characterized by the decreased abundance of the SCFA-producing bacteria including genera *Ruminococcaceae UCG-004*, *Ruminococcaceae UCG-002*, *Lachnospiraceae FCS020 group*, *Dialister* and the increased abundance of the genera in *Enterococcus* and *Enterococcaceae* with an increased production of lactic acid. Moreover, the alternation of the gut microbiota was presumably associated with the function including cell cycle control, cell division, chromosome partitioning, amino acid transport and metabolism and carbohydrate transport and metabolism through SCFA pathway. This information may not only improve our understanding of the pathogenesis of severe CHF, but also suggest that the regulation of the composition of gut microbiota may represent a promising therapeutic target.

## Data Availability Statement

The datasets presented in this study can be found in online repositories. The names of the repository/repositories and accession number(s) can be found in the article/supplementary material.

## Ethics Statement

The studies involving human participants were reviewed and approved by The First Affiliated Hospital of Harbin Medical University and The Fourth Affiliated Hospital of Harbin Medical University approved all study protocols. The patients/participants provided their written informed consent to participate in this study.

## Author Contributions

YH, PL, and HJ conceived and designed the experiments. YH and TF analyzed data and drew the pictures. WS and DD wrote this manuscript. All authors read and approved the final manuscript.

## Conflict of Interest

The authors declare that the research was conducted in the absence of any commercial or financial relationships that could be construed as a potential conflict of interest.

## Publisher’s Note

All claims expressed in this article are solely those of the authors and do not necessarily represent those of their affiliated organizations, or those of the publisher, the editors and the reviewers. Any product that may be evaluated in this article, or claim that may be made by its manufacturer, is not guaranteed or endorsed by the publisher.

## Footnotes

1
http://www.bioinformatics.babraham.ac.uk/projects/fastqc/

2
http://qiime.org/

3
https://www.arb-silva.de/

4
https://picrust.github.io/picrust/index.html

## References

[B1] Alrafas H. R. Busbee P. B. Chitrala K. N. Nagarkatti M. Nagarkatti P. (2020). Alterations in the gut microbiome and suppression of histone deacetylases by resveratrol are associated with attenuation of colonic inflammation and protection against colorectal Cancer. *J. Clin. Med.* 9:1796. 10.3390/jcm9061796 32526927 PMC7355848

[B2] Amoroso C. Perillo F. Strati F. Fantini M. C. Caprioli F. Facciotti F. (2020). the role of gut microbiota biomodulators on mucosal immunity and intestinal inflammation. *Cells* 9:1234. 10.3390/cells9051234 32429359 PMC7291275

[B3] Anker S. D. Von Haehling S. (2004). Inflammatory mediators in chronic heart failure: an overview. *Heart* 90 464–470. 10.1136/hrt.2002.007005 15020532 PMC1768165

[B4] Aponte M. Murru N. Shoukat M. (2020). Therapeutic, prophylactic, and functional use of probiotics: a current perspective. *Front. Microbiol.* 11:562048. 10.3389/fmicb.2020.562048 33042069 PMC7516994

[B5] Bhaskaran N. Quigley C. Paw C. Butala S. Schneider E. Pandiyan P. (2018). Role of short chain fatty acids in controlling tregs and immunopathology during mucosal infection. *Front. Microbiol.* 9:1995. 10.3389/fmicb.2018.01995 30197637 PMC6117408

[B6] Brial F. Le Lay A. Dumas M. E. Gauguier D. (2018). Implication of gut microbiota metabolites in cardiovascular and metabolic diseases. *Cell Mol. Life Sci.* 75 3977–3990. 10.1007/s00018-018-2901-1 30101405 PMC6182343

[B7] Byappanahalli M. N. Nevers M. B. Korajkic A. Staley Z. R. Harwood V. J. (2012). Enterococci in the environment. *Microbiol. Mol. Biol. Rev.* 76 685–706. 23204362 10.1128/MMBR.00023-12 PMC3510518

[B8] Canfora E. E. Jocken J. W. Blaak E. E. (2015). Short-chain fatty acids in control of body weight and insulin sensitivity. *Nat. Rev. Endocrinol.* 11 577–591. 10.1038/nrendo.2015.128 26260141

[B9] Cheng L. Qi C. Yang H. Lu M. Cai Y. Fu T. (2021). gutMGene: a comprehensive database for target genes of gut microbes and microbial metabolites. *Nucleic Acids Res.* gkab786. 10.1093/nar/gkab786 34500458 PMC8728193

[B10] Cheng L. Qi C. Zhuang H. Fu T. Zhang X. (2020). gutMDisorder: a comprehensive database for dysbiosis of the gut microbiota in disorders and interventions. *Nucleic Acids Res.* 48 D554–D560. 31584099 10.1093/nar/gkz843 PMC6943049

[B11] Cheng L. Yang H. Zhao H. Pei X. Shi H. Sun J. (2019). MetSigDis: a manually curated resource for the metabolic signatures of diseases. *Brief. Bioinform.* 20 203–209. 10.1093/bib/bbx103 28968812

[B12] Cui X. Ye L. Li J. Jin L. Wang W. Li S. (2018). Metagenomic and metabolomic analyses unveil dysbiosis of gut microbiota in chronic heart failure patients. *Sci. Rep.* 8:635. 10.1038/s41598-017-18756-2 29330424 PMC5766622

[B13] Dantzer R. Cohen S. Russo S. J. Dinan T. G. (2018). Resilience and immunity. *Brain Behav. Immun.* 74 28–42. 10.1016/j.bbi.2018.08.010 30102966 PMC6545920

[B14] Deledda A. Annunziata G. Tenore G. C. Palmas V. Manzin A. Velluzzi F. (2021). Diet-derived antioxidants and their role in inflammation, obesity and gut microbiota modulation. *Antioxidants* 10:708. 10.3390/antiox10050708 33946864 PMC8146040

[B15] den Besten G. Lange K. Havinga R. Van Dijk T. H. Gerding A. Van Eunen K. (2013). Gut-derived short-chain fatty acids are vividly assimilated into host carbohydrates and lipids. *Am. J. Physiol. Gastrointest. Liver Physiol.* 305 G900–G910. 10.1152/ajpgi.00265.2013 24136789

[B16] Esquivel-Rendon E. Vargas-Mireles J. Cuevas-Olguin R. Miranda-Morales M. Acosta-Mares P. Garcia-Oscos F. (2019). Interleukin 6 dependent synaptic plasticity in a social defeat-susceptible prefrontal cortex circuit. *Neuroscience* 414 280–296. 10.1016/j.neuroscience.2019.07.002 31301368

[B17] Gajarsa J. J. Kloner R. A. (2011). Left ventricular remodeling in the post-infarction heart: a review of cellular, molecular mechanisms, and therapeutic modalities. *Heart. Fail Rev.* 16 13–21. 10.1007/s10741-010-9181-7 20623185

[B18] Gao Z. Yin J. Zhang J. Ward R. E. Martin R. J. Lefevre M. (2009). Butyrate improves insulin sensitivity and increases energy expenditure in mice. *Diabetes* 58 1509–1517. 10.2337/db08-1637 19366864 PMC2699871

[B19] Gouba N. Yimagou E. K. Hassani Y. Drancourt M. Fellag M. Mbogning Fonkou M. D. (2020). *Enterococcus burkinafasonensis* sp. nov. isolated from human gut microbiota. *New Microbes New Infect.* 36:100702. 10.1016/j.nmni.2020.100702 32528688 PMC7283139

[B20] Hage C. Michaelsson E. Linde C. Donal E. Daubert J. C. Gan L. M. (2017). Inflammatory biomarkers predict heart failure severity and prognosis in patients with heart failure with preserved ejection fraction: a holistic proteomic approach. *Circ. Cardiovasc. Genet.* 10:e001633. 10.1161/CIRCGENETICS.116.001633 28100627

[B21] Han Y. Gong Z. Sun G. Xu J. Qi C. Sun W. (2021). Dysbiosis of gut microbiota in patients with acute myocardial infarction. *Front. Microbiol.* 12:680101. 10.3389/fmicb.2021.680101 34295318 PMC8290895

[B22] Harikrishnan S. (2019). Diet, the gut microbiome and heart failure. *Card Fail Rev.* 5 119–122. 10.15420/cfr.2018.39.2 31179023 PMC6545994

[B23] Huang S. Y. Xiang X. Qiu L. Wang L. Y. Zhu B. H. Guo R. Q. (2020). Transfection of TGF-beta shRNA by using ultrasound-targeted microbubble destruction to inhibit the early adhesion repair of rats wounded achilles tendon in vitro and in vivo. *Curr. Gene Ther.* 20 71–81. 10.2174/1566523220666200516165828 32416687

[B24] Iqubal A. Iqubal M. K. Khan A. Ali J. Baboota S. Haque S. E. (2020). Gene therapy, a novel therapeutic tool for neurological disorders: current progress, challenges and future prospective. *Curr. Gene Ther.* 20 184–194. 10.2174/1566523220999200716111502 32674730

[B25] Jia Q. Xie Y. Lu C. Zhang A. Lu Y. Lv S. (2019). Endocrine organs of cardiovascular diseases: gut microbiota. *J. Cell Mol. Med.* 23 2314–2323. 10.1111/jcmm.14164 30688023 PMC6433674

[B26] Kamo T. Akazawa H. Suda W. Saga-Kamo A. Shimizu Y. Yagi H. (2017). Dysbiosis and compositional alterations with aging in the gut microbiota of patients with heart failure. *PLoS One* 12:e0174099. 10.1371/journal.pone.0174099 28328981 PMC5362204

[B27] Khan A. Zahra A. Mumtaz S. Fatmi M. Q. Khan M. J. (2020). Integrated in-silico analysis to study the role of microRNAs in the detection of chronic kidney diseases. *Curr. Bioinform.* 15 144–154. 10.2174/1574893614666190923115032

[B28] Koh A. De Vadder F. Kovatcheva-Datchary P. Backhed F. (2016). From dietary fiber to host physiology: short-chain fatty acids as key bacterial metabolites. *Cell* 165 1332–1345. 10.1016/j.cell.2016.05.041 27259147

[B29] Kummen M. Mayerhofer C. C. K. Vestad B. Broch K. Awoyemi A. Storm-Larsen C. (2018). Gut microbiota signature in heart failure defined from profiling of 2 independent cohorts. *J. Am. Coll. Cardiol.* 71 1184–1186. 10.1016/j.jacc.2017.12.057 29519360

[B30] Kwon E. Cho M. Kim H. Son H. S. (2020). A study on host tropism determinants of influenza virus using machine learning. *Curr. Bioinform.* 15 121–134. 10.2174/1574893614666191104160927

[B31] Li M. Van Esch B. Henricks P. A. J. Folkerts G. Garssen J. (2018). The anti-inflammatory effects of short chain fatty acids on lipopolysaccharide- or tumor necrosis factor alpha-stimulated endothelial cells via activation of GPR41/43 and inhibition of HDACs. *Front. Pharmacol.* 9:533. 10.3389/fphar.2018.00533 29875665 PMC5974203

[B32] Long J. Yang H. Yang Z. Jia Q. Liu L. Kong L. (2021). Integrated biomarker profiling of the metabolome associated with impaired fasting glucose and type 2 diabetes mellitus in large-scale Chinese patients. *Clin. Transl. Med.* 11:e432. 10.1002/ctm2.432 34185410 PMC8167862

[B33] Lopetuso L. R. Petito V. Graziani C. Schiavoni E. Paroni Sterbini F. Poscia A. (2018). Gut microbiota in health, diverticular disease, irritable bowel syndrome, and inflammatory bowel diseases: time for microbial marker of gastrointestinal disorders. *Dig. Dis.* 36 56–65. 10.1159/000477205 28683448

[B34] Luedde M. Winkler T. Heinsen F. A. Ruhlemann M. C. Spehlmann M. E. Bajrovic A. (2017). Heart failure is associated with depletion of core intestinal microbiota. *ESC Heart Fail* 4 282–290. 10.1002/ehf2.12155 28772054 PMC5542738

[B35] Lv H. Dao F. Y. Zulfiqar H. Lin H. (2021). DeepIPs: comprehensive assessment and computational identification of phosphorylation sites of SARS-CoV-2 infection using a deep learning-based approach. *Brief. Bioinform.* 22:bbab244. 10.1093/bib/bbab244 34184738 PMC8406875

[B36] Makki K. Deehan E. C. Walter J. Backhed F. (2018). The impact of dietary fiber on gut microbiota in host health and disease. *Cell Host Microbe* 23 705–715. 10.1016/j.chom.2018.05.012 29902436

[B37] Mayerhofer C. C. K. Awoyemi A. O. Moscavitch S. D. Lappegard K. T. Hov J. R. Aukrust P. (2018). Design of the GutHeart-targeting gut microbiota to treat heart failure-trial: a Phase II, randomized clinical trial. *ESC Heart Fail* 5 977–984. 10.1002/ehf2.12332 30088346 PMC6165929

[B38] Mayerhofer C. C. K. Kummen M. Holm K. Broch K. Awoyemi A. Vestad B. (2020). Low fibre intake is associated with gut microbiota alterations in chronic heart failure. *ESC Heart Fail* 7 456–466. 10.1002/ehf2.12596 31978943 PMC7160496

[B39] Moshkelgosha S. Masetti G. Berchner-Pfannschmidt U. Verhasselt H. L. Horstmann M. Diaz-Cano S. (2018). Gut microbiome in BALB/c and C57BL/6J mice undergoing experimental thyroid autoimmunity associate with differences in immunological responses and thyroid function. *Horm. Metab. Res.* 50 932–941. 10.1055/a-0653-3766 30107619

[B40] Muller M. Hernandez M. A. G. Goossens G. H. Reijnders D. Holst J. J. Jocken J. W. E. (2019). Circulating but not faecal short-chain fatty acids are related to insulin sensitivity, lipolysis and GLP-1 concentrations in humans. *Sci. Rep.* 9:12515. 10.1038/s41598-019-48775-0 31467327 PMC6715624

[B41] Mulpuru V. Semwal R. Varadwaj P. K. Mishra N. (2021). HAMP: a knowledgebase of antimicrobial peptides from human microbiome. *Curr. Bioinform.* 16 534–540. 10.2174/1574893615999200802041228

[B42] Nadia Ramana J. (2020). The human OncoBiome database: a database of cancer microbiome datasets. *Curr. Bioinform.* 15 472–477. 10.2174/1574893614666190902152727

[B43] Nagatomo Y. Tang W. H. (2015). Intersections between microbiome and heart failure: revisiting the gut hypothesis. *J. Card Fail* 21 973–980. 10.1016/j.cardfail.2015.09.017 26435097 PMC4666782

[B44] Onyszkiewicz M. Gawrys-Kopczynska M. Konopelski P. Aleksandrowicz M. Sawicka A. Kozniewska E. (2019). Butyric acid, a gut bacteria metabolite, lowers arterial blood pressure via colon-vagus nerve signaling and GPR41/43 receptors. *Pflugers Arch.* 471 1441–1453. 10.1007/s00424-019-02322-y 31728701 PMC6882756

[B45] Pujol J. B. Christinat N. Ratinaud Y. Savoia C. Mitchell S. E. Dioum E. H. M. (2018). Coordination of GPR40 and ketogenesis signaling by medium chain fatty acids regulates beta cell function. *Nutrients* 10:473. 10.3390/nu10040473 29649104 PMC5946258

[B46] Qi C. Wang P. Fu T. Lu M. Cai Y. Chen X. (2021). A comprehensive review for gut microbes: technologies, interventions, metabolites and diseases. *Brief. Funct. Genomics* 20 42–60. 10.1093/bfgp/elaa029 33554248

[B47] Sah S. K. Kim B. H. Park G. T. Kim S. Jang K. H. Jeon J. E. (2013). Novel isonahocol E(3) exhibits anti-inflammatory and anti-angiogenic effects in endothelin-1-stimulated human keratinocytes. *Eur. J. Pharmacol.* 720 205–211. 10.1016/j.ejphar.2013.09.077 24436991

[B48] Sanchez-Rodriguez E. Egea-Zorrilla A. Plaza-Diaz J. Aragon-Vela J. Munoz-Quezada S. Tercedor-Sanchez L. (2020). The gut microbiota and its implication in the development of atherosclerosis and related cardiovascular diseases. *Nutrients* 12:605. 10.3390/nu12030605 32110880 PMC7146472

[B49] Sandek A. Bjarnason I. Volk H. D. Crane R. Meddings J. B. Niebauer J. (2012). Studies on bacterial endotoxin and intestinal absorption function in patients with chronic heart failure. *Int. J. Cardiol.* 157 80–85. 10.1016/j.ijcard.2010.12.016 21190739

[B50] Shanahan F. (2010). Probiotics in perspective. *Gastroenterology* 139 1808–1812. 10.1053/j.gastro.2010.10.025 20965190

[B51] Shin N. R. Whon T. W. Bae J. W. (2015). *Proteobacteria*: microbial signature of dysbiosis in gut microbiota. *Trends Biotechnol.* 33 496–503. 10.1016/j.tibtech.2015.06.011 26210164

[B52] Sivaprakasam S. Prasad P. D. Singh N. (2016). Benefits of short-chain fatty acids and their receptors in inflammation and carcinogenesis. *Pharmacol. Ther.* 164 144–151. 10.1016/j.pharmthera.2016.04.007 27113407 PMC4942363

[B53] Tang W. Wan S. Yang Z. Teschendorff A. E. Zou Q. (2018). Tumor origin detection with tissue-specific miRNA and DNA methylation markers. *Bioinformatics* 34 398–406. 10.1093/bioinformatics/btx622 29028927

[B54] Tang W. H. Kitai T. Hazen S. L. (2017). Gut microbiota in cardiovascular health and disease. *Circ. Res.* 120 1183–1196. 28360349 10.1161/CIRCRESAHA.117.309715 PMC5390330

[B55] Tang W. H. W. Li D. Y. Hazen S. L. (2019). Dietary metabolism, the gut microbiome, and heart failure. *Nat. Rev. Cardiol.* 16 137–154. 10.1038/s41569-018-0108-7 30410105 PMC6377322

[B56] Thevenet J. De Marchi U. Domingo J. S. Christinat N. Bultot L. Lefebvre G. (2016). Medium-chain fatty acids inhibit mitochondrial metabolism in astrocytes promoting astrocyte-neuron lactate and ketone body shuttle systems. *FASEB J.* 30 1913–1926. 10.1096/fj.201500182 26839375

[B57] Weber G. J. Foster J. Pushpakumar S. B. Sen U. (2018). Altered microRNA regulation of short chain fatty acid receptors in the hypertensive kidney is normalized with hydrogen sulfide supplementation. *Pharmacol. Res.* 134 157–165. 10.1016/j.phrs.2018.06.012 29909116 PMC6086735

[B58] Xie A. Song J. Lu S. Liu Y. Tang L. Wen S. (2021). Influence of diet on the effect of the probiotic lactobacillus paracasei in rats suffering from allergic asthma. *Front. Microbiol.* 12:737622. 10.3389/fmicb.2021.737622 34659167 PMC8516095

[B59] Yang H. Luo Y. Ren X. Wu M. He X. Peng B. (2021). Risk prediction of diabetes: big data mining with fusion of multifarious physical examination indicators. *Inform. Fusion* 75 140–149. 10.1016/j.inffus.2021.02.015

[B60] Yu Y. Jia T. Z. Cai Q. Jiang N. Ma M. Y. Min D. Y. (2015). Comparison of the anti-ulcer activity between the crude and bran-processed *Atractylodes lancea* in the rat model of gastric ulcer induced by acetic acid. *J. Ethnopharmacol.* 160 211–218. 10.1016/j.jep.2014.10.066 25481080

[B61] Yue Y. He Z. Zhou Y. Ross R. P. Stanton C. Zhao J. (2020). Lactobacillus plantarum relieves diarrhea caused by enterotoxin-producing *Escherichia coli* through inflammation modulation and gut microbiota regulation. *Food Funct.* 11 10362–10374. 10.1039/d0fo02670k 33220669

[B62] Zhang X. Y. Shi S. P. Shen J. Zhao M. Y. He Q. N. (2019). Functional immunoregulation by heme oxygenase 1 in juvenile autoimmune diseases. *Curr. Gene Ther.* 19 110–116. 10.2174/1566523219666190710092935 31288720

